# Association between triglyceride glucose index and organ involvement in patients with systemic sclerosis

**DOI:** 10.1038/s41598-025-99139-w

**Published:** 2025-05-29

**Authors:** Huidan Yang, Hao Cheng, Huifang Yang, Xiaoying Zhang, Lili Shang, Chenglan Yan, Hongyan Wen

**Affiliations:** 1 Department of Rheumatology, The First Hospital of Shanxi Medical University, Taiyuan, Shanxi China; 2 Qinshui County People’s Hospital, Jincheng, Shanxi China; 3 Department of Rheumatology, The Second Hospital of Shanxi Medical University, Taiyuan, Shanxi China

**Keywords:** Triglyceride-glucose index, Systemic sclerosis, Cardiovascular disease, Medical research, Rheumatology

## Abstract

Patients with systemic sclerosis (SSc) exhibit an elevated risk of multi-organ involvement. Vasculopathy, a hallmark pathological feature of SSc, is closely related to cardiac and renal complications. The triglyceride glucose (TyG) index shows a strong association with vascular injury. In this study, we aimed to investigate the relationship between the TyG index and SSc-related organ involvement and assess its potential predictive value for cardiovascular disease risk. This retrospective cross-sectional study of 102 patients with systemic sclerosis and 89 age- and sex-matched healthy controls were enrolled. The TyG index was calculated by ln [fasting triglycerides (mg/dl) * fasting glucose (mg/dl)/2]. All systemic sclerosis patients were grouped according to the TyG quartile and whether combined with cardiovascular disease. The association between TyG and cardiovascular disease occurrence was evaluated by multivariate regression analysis, restricted cubic spline (RCS), and subgroup analysis. Receiver operating characteristic curves assessed the predictive value of TyG index. Mean TyG index was higher in systemic sclerosis patients versus controls (8.50 $\:\pm\:$ 0.45 vs. 8.33 $\:\pm\:$ 0.43; *P* = 0.006). Systemic sclerosis patients with cardiovascular disease or renal involvement had higher TyG index than those without [(8.69 $\:\pm\:$ 0.42) vs. (8.39 $\:\pm\:$ 0.43), *P* = 0.001; (8.65 $\:\pm\:$ 0.44) vs. (8.45 $\:\pm\:$ 0.41), *P* = 0.044], while patients with joint involvement had lower TyG index [(8.38 $\:\pm\:$ 0.44) vs. (8.59 $\:\pm\:$ 0.43), *P* = 0.019]. After grouping according to the TyG quartiles, the incidence of cardiovascular disease and modified Rodnan skin score (mRSS) gradually increased from Q1 to Q4. TyG showed a positive correlation with mRSS score (*r* = 0.307, *P* = 0.002). High TyG index was an independent risk factor for cardiovascular disease (OR = 4.03, 95% CI = 1.07–15.10, *P* = 0.039). TyG index had moderate predictive value for the risk of cardiovascular disease. The TyG index is associated with skin fibrosis and cardiovascular risk in systemic sclerosis and may serve as an effective biomarker for cardiovascular disease risk assessment.

## Introduction

Systemic sclerosis (SSc) is an autoimmune disease characterized by fibrosis of multiple organs. According to the range of skin involvement and clinical characteristics, SSc can be divided into 4 types: limited cutaneous systemic sclerosis (lcSSc), diffuse cutaneous systemic sclerosis (dcSSc), overlap syndrome (limited or diffuse cutaneous SSc coexisting with other connective tissue diseases), and SSc sine scleroderma. Common manifestations include Raynaud’s phenomenon, skin sclerosis, interstitial lung disease (ILD), cardiovascular and gastrointestinal involvement, renal and joint abnormalities^1^. SSc has a prevalence of 17.6 per 100,000^2^. Insulin resistance (IR) is more common in systemic autoimmune diseases like rheumatoid arthritis and systemic lupus erythematous^3–5^. IR can contribute to atherosclerosis and cardiovascular disease (CVD)^6,7^. A few studies suggest IR is also more prevalent in SSc, IR contributes to endothelial dysfunction, a key feature in SSc vasculopathy, which can lead to digital ulcers (DUs), pulmonary arterial hypertension (PAH) and CVD^4,8,9^. DUs are associated with skin fibrosis in SSc. Assessing IR may help to prevent future complications in SSc.

The triglyceride glucose (TyG) index, calculated as ln [triglycerides (mg/dl) * glucose (mg/dl)/2], is a simple surrogate marker of IR, correlating with gold standard measures like hyperinsulinemic-euglycemic clamp^10,11^. Moreover, unlike HOMA-IR, which requires insulin assays, the TyG index is easily accessible and practical for routine clinical use, which can be developed in primary hospitals. Higher TyG indexes are associated to diabetes, hypertension, CVD events, and chronic kidney disease progression^12–14^. However, no studies have examined associations between TyG index and organ involvement in SSc.

Therefore, this study aimed to explore the association between TyG index and organ involvement in SSc patients, this may be a novel biomarker for the early identification of CVD. These problems will provide a handy reference for the prevention and treatment in the clinical practice.

## Materials and methods

### Patients and study design

A total of 102 SSc patients were enrolled, who visited the Rheumatology and Immunology Department of the Affiliated Hospital of Shanxi Medical University from January 2018 to March 2023. The inclusion criteria were as follows: (i) met the American College of Rheumatology/European League Against Rheumatism (ACR/EULAR) classification with confirmatory diagnosis of SSc^15^; (ii) available complete case information. We excluded participants who: (i) had a combination of diabetes, liver insufficiency, acute infection, and presence of tumors; (ii) were treated with triglyceride-lowering drugs (e.g. fibrates, niacin or omega-3) or glucose-lowering agents which may influence the key indicator TyG, thus confounding the results. In addition, these drugs may directly or indirectly affect CVD and fibrosis occurrence. We also included 89 age- and sex- matched healthy controls (HCs) without history of rheumatic immune system disease or family history, who were received physical examination in our hospital Physical Examination Center. Our study was approved by the Ethics Committee of the Shanxi Medical University Affiliated Hospital (2022YX009) and the written informed consent was waived by the institutional ethics committee of The Shanxi Medical University Affiliated Hospital because of the retrospective observational nature of the study. We confirm that all methods were performed in accordance with the relevant guidelines and regulations.

### Data collection and measurements

The basic demographic and clinical characteristics of the study population were recorded including sex, age, body mass index (BMI), duration of SSc, disease classification, smoking status, alcohol consumption, and primary symptoms.

Venous blood samples were collected from all patients after an overnight fast on the second morning following admission. Laboratory analyses included fasting blood glucose (FBG), triglycerides (TG), total cholesterol (TC), low-density lipoprotein cholesterol (LDL-C), high-density lipoprotein cholesterol (HDL-C), alanine aminotransferase (ALT), aspartate aminotransferase (AST), C-reactive protein (CRP), erythrocyte sedimentation rate (ESR), blood urea nitrogen (BUN), creatinine (Cr), blood uric acid (UA), and SSc-related serological markers including anti-nuclear antibodies (ANA), anti-centromere antibodies (ACA), and anti-Scl-70 antibodies. BMI was calculated as weight (kilograms)/height squared (in meters), and the TyG index was derives using the formula ln [TG (mg/dl) × FBG (mg/dl)/2].

Organ involvement assessments were conducted by specialists. Raynaud’s phenomenon and digital ulcers (DUs) were defined by clinical manifestations. Pulmonary arterial hypertension (PAH) refers to a pulmonary artery pressure > 30mmHg measured by cardiac color ultrasound. ILD refers to the symptoms of cough, tight breath, and HRCT showed ground glass shadow, lobular interval thickening, grid shading, honeycomb sign, or traction bronchiectasis, and pulmonary function tests (PFTs) shows FVC (forced vital capacity) < 80% predicted and/or DLCO (diffusing capacity for carbon monoxide) < 70%. CVD encompassed hypertension, coronary heart disease (CHD), arrhythmia, and heart failure (HF). Renal involvement was defined as persistent proteinuria > 500 mg/day or serum creatinine level > 30% above baseline. Joint involvement was characterized by arthralgia or reduced mobility. Digestive system involvement includes dysphagia, gastroesophageal reflux, malabsorption, and etc. Skin fibrosis severity was quantified using the modified Rodnan skin score (mRSS), where 17 body areas are graded 0–3 (maximum score = 51), with higher scores indicating more severe involvement.

### Statistical analysis

All data were analyzed using IBM SPSS Statistics version 25.0, GraphPad Prism 9, R(4.4.1), along with Zstats v0.90 (www.medsta.cn/software). Normally distributed data are expressed as the mean ± standard deviation, nonnormal distributions are expressed as the median and quartiles, and count data were expressed as percentages and absolute frequencies. Differences in continuous variables were compared using t-tests or Mann-Whitney U tests. For the analysis of continuous variables among multiple groups, one-way ANOVA and the Kruskal-Wallis H test were used. While categorical variables were compared using chi-square tests. Correlation analysis was performed using Spearman’s correlation. Logistic regression analyzed independent risk factors for CVD, expressed as the odds ratio (OR) and 95% CI. Logistic models adjusted for known traditional risk factors. Model I was adjusted for sex, age, and BMI. Model II was adjusted for sex, age, BMI, HDL-C, white blood cells, lymphocyte, Cr, and UA. Adjusting for HDL-C is crucial, as low HDL-C levels are independently associated with increased cardiovascular risk in SSc^16^. Restricted cubic spline (RCS) and subgroup analysis were used to assess the association between TyG and cardiovascular disease occurrence. Plotting the receiver operating characteristic curve (ROC), and reporting the area under the curve (AUC) evaluate the predictive ability of the TyG index for CVD in SSc. The statistical tests were two sided, with *P* < 0.05 as being significant.

## Results

### Characteristics of the study participants

**Fig. 1:**
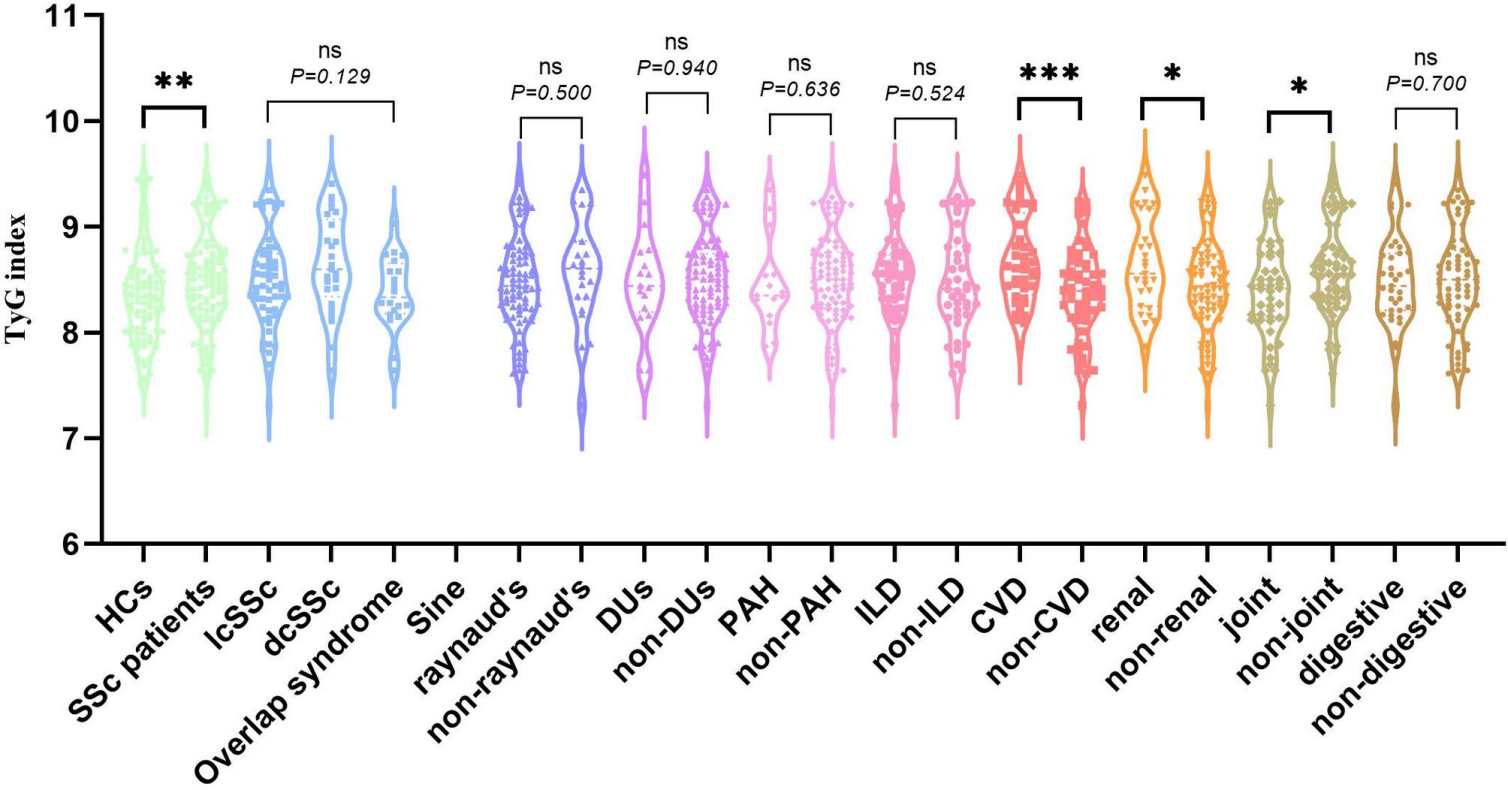
The level of TyG index in patients with SSc, stratified by disease classification, Raynaud’s phenomenon, DUs, PAH, ILD, CVD, renal, joint, and digestive involvement(*P < 0.05, **P < 0.01, ***P < 0.001). (TyG index, triglyceride glucose index; SSc, systemic sclerosis; DUs, digital ulcers; PAH, pulmonary arterial hypertension; ILD, interstitial lung disease; CVD, cardiovascular disease. )

Mean TyG index was higher in SSc patients versus controls (8.50 $\:\pm\:$ 0.45 vs. 8.33 $\:\pm\:$ 0.43; *P* = 0.006) (Table 1). SSc patients with renal involvement or CVD had higher TyG index than those without [(8.65 $\:\pm\:$ 0.44) vs. (8.45 $\:\pm\:$ 0.44), *P* = 0.044; (8.69 $\:\pm\:$ 0.42) vs. (8.39 $\:\pm\:$ 0.43), *P* = 0.001], while patients with joint involvement had lower TyG index [(8.38 $\:\pm\:$ 0.44) vs. (8.59 $\:\pm\:$ 0.43), *P* = 0.019] (Fig. 1). There were no statistical differences between with and without raynaud’s phenomenon, DUs, PAH, ILD and digestive system involvement [(8.49 $\:\pm\:$ 0.43) vs. (8.56 $\:\pm\:$ 0.49), *P* = 0.500; (8.50 $\:\pm\:$ 0.49) vs. (8.50 $\:\pm\:$ 0.44), *P* = 0.940; (8.45 $\:\pm\:$ 0.43) vs. (8.51 $\:\pm\:$ 0.45), *P* = 0.636; (8.53 $\:\pm\:$ 0.43) vs. (8.47 $\:\pm\:$ 0.47), *P* = 0.524; (8.48 $\:\pm\:$ 0.42) vs. (8.52 $\:\pm\:$ 0.46), *P* = 0.700]. In the subgroup analysis, dcSSc patients had a higher mean TyG index compared to lcSSc patients, but there was no statistically significant difference in TyG index between 57 lcSSc, 25 dcSSc and 20 overlap syndrome [(8.49 $\:\pm\:$ 0.47) vs. (8.64 $\:\pm\:$ 0.46) vs. (8.38 $\:\pm\:$ 0.34), *P* = 0.129].

**Table 1:** Comparision of the basic information in SSc patients and healthy controls.

Characteristics	Age (years)	Female (%)	BMI (Kg/m^2^)	TG (mmol/L)	FBG (mmol/L)	TyG index
SSc patients (*n* = 102)	53.43$\:\:\pm\:$ 13.52	90(88.24)	21.50 ± 3.58	1.31(1.04, 1.77)	4.73 $\:\pm\:$ 0.73	8.50 $\:\pm\:$ 0.45
HCs (*n* = 89)	51.11$\:\:\pm\:$ 6.00	72(80.90)	21.56 ± 3.81	1.05(0.79, 1.35)	4.87 $\:\pm\:$ 0.52	8.33 $\:\pm\:$ 0.43
*P-*values	0.120	0.159	0.260	0.001	0.128	0.006

HCs, healthy controls; BMI, body mass index; TG, triglycerides; FBG, fasting blood glucose; TyG index, triglyceride glucose index.

### Organ involvement in different TyG categories

After grouping according to the TyG quartiles, significant differences in the incidence of the CVD and mRSS were observed between the different categories (*P* < 0.05, Table 2). In Q1 to Q4, the incidence of CVD and mRSS gradually increased. The incidence of CVD in the Q4 category was as high as 60.0%. TyG showed a positive correlation with mRSS (*r* = 0.307, *P* = 0.002). Although the correlation coefficient (*r* = 0.307) between TyG and mRSS suggests only a mild association, this may still hold clinical significance in identifying early fibrotic changes. In Q3 and Q4, the incidence of renal involvement increased, but no statistical difference between the different categories (*P* > 0.05). The incidence of joint involvement gradually decreased, but there was no significant difference (*P* > 0.05). There were no significant differences in with and without raynaud’s phenomenon, DUs, PAH, pulmonary, and digestive system involvement (*P* > 0.05).

**Table 2:** Organ involvement for SSc patients in different TyG categories.

	Overall	Q1 (TyG ≤ 8.23)	Q2 (8.23< TyG ≤ 8.49)	Q3 (8.49< TyG ≤ 8.75)	Q4 (TyG > 8.75)	*P*-values
Number	102	26	25	26	25	-
Raynaud’s (%)	80(78.4)	21(80.8)	21(84.0)	19(73.1)	19(76.0)	0.784
DUs (%)	17(16.7)	5(19.2)	4(16.0)	3(11.5)	5(20.0)	0.836
PAH (%)	15(14.7)	4(15.4)	6(24.0)	2(7.7)	3(12.0)	0.414
CVD (%)	40(39.2)	6(23.1)	8(32.0)	11(42.3)	15(60.0)	0.046
ILD (%)	60(58.8)	14(53.8)	12(48.0)	21(80.8)	13(52.0)	0.068
Renal (%)	27(26.4)	6(23.1)	4(16.0)	7(26.9)	10(40.0)	0.271
Joint (%)	44(43.1)	16(61.5)	11(44.0)	8(30.8)	9(36.0)	0.125
Digestive (%)	35(34.3)	10(38.5)	8(32.0)	9(34.6)	8(32.0)	0.957
mRSS (score)	8.0(5.0, 14.5)	6.0(3.5, 12.0)	8.0(2.0, 11.5)	9.5(6.0, 15.0)	14.0(8.0, 22.5)	0.008

TyG index, triglyceride glucose index; SSc, systemic sclerosis; DUs, digital ulcers; PAH, pulmonary arterial hypertension; CVD, cardiovascular disease; ILD, interstitial lung disease; mRSS, modified Rodnan skin score.

### Demographic and clinical characteristics in SSc patients with and without CVD

We analyzed the basic information of the SSc, as shown in Table 3. In the groups with and without CVD, there was no difference in sex, BMI, drinking, smoking, and duration, the levels of TC, LDL-C, FBG, hemoglobin, platelets, ESR, CRP, ALT, AST, BUN, ANA, ACA, and Anti-Scl-70 antibodies were also no different (all *P* > 0.05). Combined CVD SSc patients were older, had higher TyG index, TG, white blood cells, Cr and UA, and lower levels of HDL-C and Lymphocyte, the differences were statistically significant (all *P* < 0.05).

**Table 3:** Comparision of the demographic and clinical characteristics in SSc patients with and without CVD.

	Total (*n* = 102)	CVD (*n* = 40)	Non-CVD (*n* = 62)	*P*-values
Sex [male, n (%)]	12 (11.8)	5(12.5)	7(11.3)	1.000
Age (years)	53.43$\:\:\pm\:$ 13.52	57.20$\:\:\pm\:$ 11.85	51.00 $\:\pm\:$ 14.05	0.023
BMI (Kg/m2 )	21.50$\:\:\pm\:$ 3.58	21.24 $\:\pm\:$ 3.64	21.66 $\:\pm\:$ 3.57	0.563
Drinking [n (%)]	6 (5.9)	3(7.5)	3(4.8)	0.899
Somking [n (%)]	14 (13.7)	7(17.5)	7(11.3)	0.374
Duration of SSc (years)	4.00(1.00,10.00)	7.50(1.00,10.00)	3.00(1.00,10.00)	0.224
TyG index	8.50 $\:\pm\:$ 0.45	8.69 $\:\pm\:$ 0.42	8.39 $\:\pm\:$ 0.43	0.001
TG (mmol/L)	1.31(1.04,1.77)	1.44(1.19,2.16)	1.18(0.85,1.56)	0.002
TC (mmol/L)	4.27$\:\:\pm\:$ 1.11	4.00 $\:\pm\:$ 1.04	4.44 $\:\pm\:$ 1.12	0.050
HDL-C (mmol/L)	1.17(0.92,1.49)	1.07(0.82,1.29)	1.30(1.09,1.54)	0.002
LDL-C (mmol/L)	2.33$\:\:\pm\:$ 0.69	2.21 $\:\pm\:$ 0.64	2.41 $\:\pm\:$ 0.72	0.150
FBG (mmol/L)	4.73$\:\:\pm\:$ 0.73	4.76 $\:\pm\:$ 0.82	4.71 $\:\pm\:$ 0.67	0.718
White blood cells (×10^9^/L)	6.28(4.62,7.91)	7.43(4.80,9.90)	5.83(4.44,7.28)	0.019
Hemoglobin (g/L)	126.50(113.00,136.00)	122.50(109.00,137.50)	128.00(115.50,135.25)	0.322
Platelets (×10^9^/L)	206.00(172.75,271.25)	192.50(151.50,266.75)	209.00(174.75,274.75)	0.234
Lymphocyte(×10^9^/L)	1.39(1.00,1.92)	1.10(0.73,1.61)	1.65(1.18,1.97)	0.001
ESR (mm/h)	21.50(12.75,40.00)	19.50(10.25,42.25)	22.50(14.50,40.00)	0.984
CRP (mg/dL)	3.61(1.99,12.08)	5.83(2.43,16.79)	3.14(1.85,8.79)	0.131
ALT (U/L)	17.00(12.58,28.10)	18.95(12.50,39.23)	15.55(12.58,25.80)	0.202
AST (U/L)	22.65(18.05,32.18)	23.60(19.33,37.83)	21.15(17.35,27.08)	0.165
BUN (mmol/L)	4.90(3.78,6.29)	5.08(4.00,9.10)	4.65(3.67,5.80)	0.068
Cr (umol/L)	56.00(47.78,64.00)	60.50(48.25,87.50)	54.50(46.00,61.25)	0.022
UA (umol/L)	273.00(234.25,326.25)	291.62(241.00,387.75)	264.50(223.25,302.25)	0.020
ANA [n (%)]	84 (77.5)	35(87.5)	54(87.1)	0.952
ACA [n (%)]	19(18.6)	8(20.0)	11(17.7)	0.775
Anti-Scl-70 [n (%)]	30(29.4)	9(22.5)	21(33.9)	0.218

TyG index, triglyceride glucose index; CVD, cardiovascular disease; BMI, body mass index; TG, triglycerides; TC, total cholesterol; LDL-C, low-density lipoprotein cholesterol; HDL-C, high-density lipoprotein cholesterol; FBG, fasting blood glucose; ESR, erythrocyte sedimentation rate; CRP, C-reactive protein; ALT, alanine aminotransferase; AST, aspartate aminotransferase; BUN, blood urea nitrogen; Cr, creatinine; UA, blood uric acid; ANA, anti-nuclear antibodies; ACA, anti-centromere antibodies.

### Logistic regression analyse

The results of the logistic regression analysis are shown in Table 4. It was found that TyG was significantly associated with the risk of CVD, with an OR (95% CI) of 5.43(1.90-15.53). OR = 5.43 indicates that high TyG is a risk factor for CVD development, and the risk is more than five times that of normal patients. After adjusting for potential confounding factors, such as sex, age, BMI, HDL-C, white blood cells, lymphocyte, Cr, and UA, the significant association between increased TyG and the risk of CVD still existed (OR = 4.03, 95% CI = 1.07–15.10, *P* = 0.039). OR = 4.03 suggests that high TyG is still a risk factor for CVD after controlling for other factors.

**Table 4:** Logistic regression analysis of the association between the TyG and the risk of cardiovascular disease.

	OR(95% CI)	*P*-value
Unadjusted Model	5.43(1.90−15.53)	0.002
Adjusted Model 1	5.53(1.87–16.41)	0.002
Adjusted Model 2	4.03(1.07–15.10)	0.039

Model 1: Adjusted for sex, age, and BMI; Model 2: Adjusted for sex, age, BMI, HDL-C, white blood cells, lymphocyte, Cr, and UA.

### Association between TyG and the risk of CVD

**Fig. 2:**
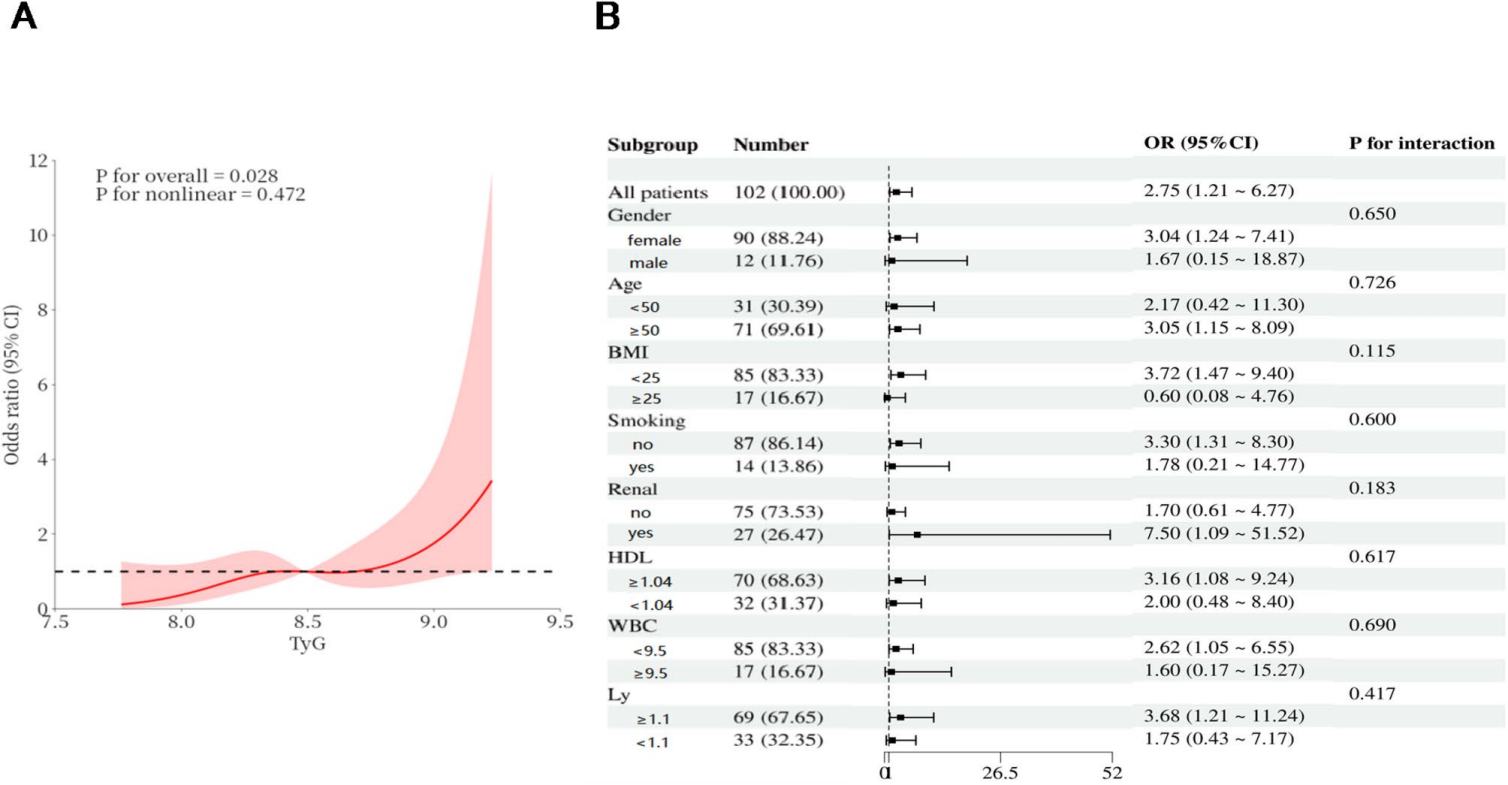
(A) The linear correlation between TyG and the risk of CVD. (B) The forest plots of the subgroup analysis detecting the impact of TyG > 8.49 on risk of CVD in different subgroups. (BMI, body mass index; HDL-C, high-density lipoprotein cholesterol; WBC, white blood cells; Ly, lymphocyte; TyG index, triglyceride glucose index.)

Figure 2A shows the relationship between TyG and the risk of CVD using RCS curves. The curve revealed a linear correlation between TyG and the risk of CVD in SSc patients (*P*-overall = 0.028, *P*-nonlinear = 0.472). The optimal cut-off value is 8.49. The results support that TyG > 8.49 was significantly associated with the risk of CVD (ORs > 1). Figure 2B presents the forest plot of the subgroup analysis. Additionally, there was no significant interaction effect between stratified by gender, age, BMI, smoking, renal involvement, HDL, WBC and Ly between TyG and the risk of CVD.

### Potential predictive value of the TyG index for the risk of CVD

**Fig. 3:**
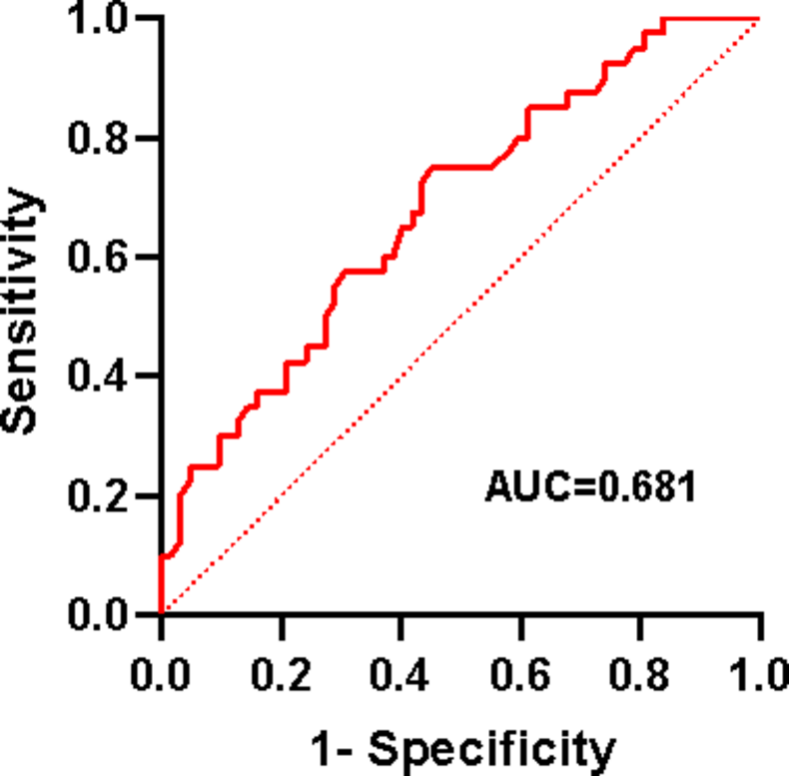
Diagnostic efficacy of TyG for CVD in SSc patients.

We further analyzed the predictive power of TyG index for the risk of CVD. Using TyG index to plot ROC curve, and report the area under the curve (AUC). An AUC greater than 0.5 is considered to have diagnostic value. The results are shown in Fig. 3. The AUC was 0.681 (95% CI = 0.576–0.786).

## Discussion

In this study, we report for the first time that TyG index was significantly associated with organ involvement in SSc patients, including renal involvement, CVD, and joint involvement. After stratification by TyG quartiles, the incidence of CVD progressively increased from Q1 to Q4. Subsequently, we compared demographic and clinical characteristics between SSc patients with and without CVD, adjusting for potential confounders in logistic regression analysis. The results revealed SSc patients with cardiovascular involvement had substantially higher TyG index than those without [(8.69 $\:\pm\:$ 0.42) vs. (8.39 $\:\pm\:$ 0.43), *P* = 0.001], with a high TyG index identified as an independent risk factor for cardiovascular involvement in SSc (OR = 4.03, 95% CI = 1.07–15.10, *P* = 0.039). Wang et al.^9^ similary demonstrated that an elevated TyG index independently predicts cardiovascular events in RA patients, corroborating our findings in SSc. CVD remains a leading cause of mortality in SSc; according to 2010 EUSTAR database data, 26% of SSc-related deaths were attributed to cardiac causes (primarily heart failure and arrhythmias)^17^. However, the pathogenesis of CVD in SSc remains unclear, as traditional risk factors fail to fully explain its occurrence^16^. A recent study by Conrad et al.^18^ reported a hazard ratio of 3.59 for CVD in SSc, further supporting our observations.

The TyG index, calculated as ln [fasting triglycerides (mg/dl) * fasting glucose (mg/dl)/2], strongly correlates with HOMA-IR and Clamp^10,11^. Therefore, TyG index is a simple and credible surrogate marker of IR. IR refers to a state of reduced responsiveness of insulin-targeting tissues to physiological levels of insulin, eventually leading to disorder of glucose and lipid metabolism, endothelial dysfunction, and arterial stiffness, and impairment of multiple organ functions including the heart and liver^19^. In rheumatic autoimmune diseases, high inflammatory cytokines TNF-α and IL-6 promote IR by inhibiting insulin signaling^20^. Emerging evidence highlights elevated IR and metabolic syndrome prevalence in systemic connective tissue diseases such as RA and SLE, which may accelerate atherosclerosis and amplify cardiovascular risk^3,21–23^. For instance, a Mexican cross-sectional study of 95 female RA and 57 SLE patients utilized the TyG index to screen for IR, revealing that approximately half of these patients exhibited IR^24^. Besides, Park et al.^4^ reported significantly elevated HOMA-IR and triglyceride levels in SSc patients compared to healthy controls, which may drive endothelial dysfunction and digital ulcer development.

Our study also identified a positive correlation between the TyG index and mRSS, suggesting its potential utility in detecting early fibrotic progression. However, specific mechanisms still need to be further explored. A cross-sectional study based on US adults showed that TyG-related parameters [triglyceride glucose-waist circumference (TyG-WC), triglyceride glucose-waist-to-height ratio (TyG-WHtR), and triglyceride glucose-body mass index (TyG-BMI)] may link to liver fibrosis^25^. Under IR and inflammatory conditions, macrophages and hepatic stellate cells are activated to promote extracellular matrix deposition and tissue remodeling to accelerate fibrosis. IR may exacerbate fibrosis via TGF-β pathway activation, culminating in fibroblast proliferation and extracellular matrix accumulation—key features of SSc pathogenesis.

To our knowledge, this is the first study to demonstrate a linear association between the TyG index and organ involvement especially CVD and skin fibrosis in SSc patients. The TyG index exhibits moderate predictive value for CVD risk stratification in this population. Given its simplicity, accessibility, and strong correlation with IR, the TyG index could be incorporated into routine SSc monitoring protocols, especially in primary care settings.

However, several limitations must be acknowledged. First, this single-center retrospective study had a limited sample size and potential selection bias (e.g., ethnic homogeneity). Exclusion of patients on triglyceride-lowering therapies may underestimate the IR-SSc association and limits generalizability to this subgroup. Second, our cohort predominantly comprised mild-to-moderate SSc cases, as critically ill patients often seek care at tertiary centers, resulting in underrepresentation of severe complications (e.g., digital ulcers, scleroderma renal crisis). Third, some organ involvement definitions lacked specificity: renal involvement criteria included secondary nephropathies (e.g., hypertension-related kidney damage), and PAH screening relied on echocardiography (a non-invasive surrogate for right heart catheterization), which may misclassify borderline cases. Future multicenter prospective studies with larger, diverse cohorts, standardized organ involvement criteria, and longitudinal follow-up are needed to validate these findings.

## Conclusion

Our findings suggest that the TyG index is associated with cardiovascular risk in SSc patients and may aid in identifying organ involvement. While promising, external validation is required before clinical implementation. Owing to its practicality, the TyG index could serve as a cost-effective tool for risk assessment in SSc management, particularly in primary care.

## Data Availability

The datasets used and/or analysed during the current study are available from the corresponding author on reasonable request.

## Abbreviations

SSc: Systemic sclerosis
ILD: Interstitial lung disease
CVD: Cardiovascular disease
TyG: Triglyceride glucose
FBG: Fasting blood glucose
TG: Triglycerides
TC: Total cholesterol
LDL-C: Low-density lipoprotein cholesterol
HDL-C: High-density lipoprotein cholesterol
ALT: Alanine aminotransferase
AST: Aspartate aminotransferase
ESR: Erythrocyte sedimentation rate
CRP: C-reactive protein
BUN: Blood urea nitrogen
Cr: Creatinine
UA: Blood uric acid
mRSS: Modified Rodnan Skin Score
